# Gender Differences in the Effect of Facial Attractiveness on Perception of Time

**DOI:** 10.3389/fpsyg.2019.01292

**Published:** 2019-06-04

**Authors:** Yu Tian, Lingjing Li, Huazhan Yin, Xiting Huang

**Affiliations:** ^1^School of Psychology, Southwest University, Chongqing, China; ^2^Key Research Base of Humanities and Social Sciences, Southwest University, Chongqing, China; ^3^The Experimental Middle School Attached to Yunnan Normal University, Kunming, China; ^4^Cognition and Human Behavior Key Laboratory of Hunan Province, Hunan Normal University, Changsha, China

**Keywords:** time perception, facial attractiveness, gender difference, esthetic standards, temporal reproduction task

## Abstract

Time perception plays a fundamental role in human social activities, and it can be influenced in social situations by various factors, including facial attractiveness. However, in the eyes of observers of different genders, the attractiveness of a face varies. The current study aimed to explore whether gender modulates the effect of facial attractiveness on time perception. To account for individual differences in esthetic standards, the critical stimuli presented to each participant were selected from an image pool based on the participant’s own attractiveness judgments. In Experiment 1, men and women performed a stimuli selection task followed by a temporal reproduction task to measure their time perception of faces of different attractiveness levels and gender. To control for the potential influence of task order, Experiment 2 flipped the order of the selection and temporal tasks. Taken together, the experiments showed that both men and women exhibited longer reproduced durations for attractive opposite-sex faces than for unattractive opposite-sex faces; conversely, in the same-sex face condition, women still exhibited longer reproduced durations for attractive faces than for unattractive faces, whereas the effect of facial attractiveness on time perception among men tended to be smaller or even fail to reach significance. These results suggest that gender differences play an important role in the effect of facial attractiveness on time perception.

## Introduction

Albert Einstein said, “Put your hand on a hot stove for a minute, and it seems like an hour. Sit with a pretty girl for an hour, and it seems like a minute.” Although Einstein’s purpose is to illustrate “relativity,” it also reflects a phenomenon wherein people’s time perception is not stable, and how attractiveness can modulate it.

In empirical research, attractive faces are often used to manipulate attractiveness. Moreover, some researchers have recruited women as participants to examine the effect of attractiveness on time perception. For example, Ogden (2013) used images of female faces to investigate how attractiveness affected the time perception of women. The facial images were presented to participants for 124, 348, 582, 767, 958, and 1,183 ms, and the participants verbally estimated how long each image lasted in milliseconds after a delay ranging from 1,000 to 1,500 ms. Participants underestimated the duration of display of unattractive female faces relative to that of attractive and neutral faces. In another study, Arantes et al. (2013) presented images of both male and female faces to women for 133, 233, 300, 383, 533, 1,050, and 2,100 ms, and then instructed them to reproduce the duration of each image. They found that participants’ reproduced durations for attractive male faces were longer than were their corresponding estimates for unattractive male faces, whereas there was no significant difference in women’s estimated durations for attractive and unattractive female faces. Tomas and Španić (2016) explored how facial expression and attractiveness interact with time perception. They presented facial images to participants for 400 to 1,600 ms and asked them to determine whether the duration of each image was more similar to the short (400 ms) or long anchor duration (1,600 ms). Participants tended to judge the display duration of angry faces as longer compared to neutral ones, but only when the faces were attractive; the effect did not occur in the case of unattractive faces. It seems that there is an effect of facial attractiveness on time perception despite the inconsistent results.

In the field of time psychology, temporal variation can be explained by Scalar Expectancy Theory (SET), which was developed from the internal clock model (Treisman, 1963). SET assumes the existence of an internal clock/pacemaker-accumulator device whereby time is measured according to the number of pulses generated by an arousal-related pacemaker and counted by an accumulator through the closing of an attention-controlled switch (Gibbon et al., 1984). An increase in arousal is generally associated with acceleration of the pacemaker. When attention is oriented toward timing, the switch closes and pulses pass into the accumulator. The pulses are blocked when attention is oriented away from timing. Alternatively, Zakay and Block (1997) created the Attentional-Gate Model (AGM) to explain the role of attention in timing. AGM proposes that an attentional gate is used instead of the switch seen in SET. Unlike this switch, which once closed is thought to remain closed throughout a given instance of timing, the gate in the AGM can open and close throughout the timing. The extent to which the gate closes is determined by the amount of attention allocated to the timing. There is empirical evidence for the effect of arousal and attention on time perception. For example, researchers who measured arousal through both psychophysiological response and subjective rating found that temporal dilating is related to increasing arousal (Angrilli et al., 1997; Mella et al., 2011; van Hedger et al., 2017; Piovesan et al., 2018). Furthermore, stimuli that capture attention early may result in a longer time perception than other stimuli (Grommet et al., 2011). Researchers who manipulated the allocation of attention have observed that the more attention is allocated to timing, the longer the perceived time (Macar et al., 1994; Chen et al., 2007; Matthews et al., 2012). Thus, arousal and attention are considered to be the two main determinants of time perception (Lake et al., 2016).

Additionally, empirical research has shown that facial attractiveness might modulate arousal in women. Attractive faces evoke a significant facial electromyographic response relative to unattractive faces (Hazlett and Hoehn-Saric, 2000). The activation of reward-related areas is greater when viewing attractive faces compared to viewing unattractive faces, although the amygdala response to both attractive and unattractive faces is undifferentiated (Liang et al., 2010). Attractive faces elicit a larger early posterior negativity and late parietal positivity, both of which are associated with higher arousal, compared to unattractive faces (Werheid et al., 2007; Paulmann et al., 2013). Furthermore, facial attractiveness has also been shown to modulate attention in women. Attractive faces capture more attention than do unattractive faces (Sui and Liu, 2009; Valuch et al., 2015). Attractive faces are also more effectively tracked than are unattractive faces (Liu and Chen, 2012), even when the low-level properties of the faces (i.e., luminance, contrast, and color saturation) are equalized (Li et al., 2016). Therefore, women’s subjective perception of the duration for which an attractive or unattractive face appears might differ as a result of these variations in arousal and attention.

Although various aspects of the stimuli and experimental settings may have contributed to the inconsistent results described above (Arantes et al., 2013; Tomas and Španić, 2016), we believe that the failure to account for individual differences in esthetic preferences is critical. Specifically, previous manipulations of attractiveness have been based on average ratings of attractiveness, in which researchers presented images with high average ratings in attractive face conditions, and those with low average ratings in unattractive face conditions; thus, the same materials were presented to each participant. Although individuals’ standards for facial esthetics have much in common, there is variation in these standards among observers arising from biological, psychological, behavioral, and social factors (Cunningham et al., 1995; Kou et al., 2013). Thus, these previous researchers might have failed to manipulate attractiveness effectively, especially those who only presented five images, six images, or a single image in each attractiveness condition (Arantes et al., 2013; Ogden, 2013; Tomas and Španić, 2016). Thus, previous manipulations of attractiveness might have introduced instability into the results.

Furthermore, previous researchers have focused on perception in women and ignored men. Some evidence suggests that there are many gender differences in non-verbal decoding (Hall, 1978, 1990; Hall and Gunnery, 2013; Fischer et al., 2018). There is some evidence that the observer’s gender may modulate the effect of facial attractiveness on time perception. For example, in the domain of arousal, researchers have observed that an arousal-related neural region (e.g., orbito-frontal cortex) is more active when men viewed attractive female faces than when women viewed attractive male faces (Cloutier et al., 2008). Attractive female faces evoke stronger arousal-related electroencephalographic activation (i.e., late positive component) than do unattractive female faces in men, while a similar effect is not observed in women (Zhang et al., 2012). In the domain of attention, although both men and women have attentional bias to attractive faces (Aharon et al., 2001; Iaria et al., 2008), men show a stronger bias than do women toward attractive opposite-sex faces. That is, attractive female faces capture and retain men’s attention more effectively than do attractive male faces, whereas women exhibit no attentional bias toward opposite-sex attractive faces (Maner et al., 2003; Valuch et al., 2015). It is reasonable to hypothesize that the effect of facial attractiveness on time perception may differ between men and women according to SET.

To explore whether gender modulates the effect of facial attractiveness on time perception, we recruited both men and women to complete a temporal measurement task while viewing attractive female faces, attractive male faces, unattractive female faces, and unattractive male faces as stimuli. Given individual differences in esthetic standards, we assembled a pool of images of male and female faces of varying levels of attractiveness, and asked each participant to select several attractive and unattractive same-sex and opposite-sex faces as their personal stimuli. Although many types of temporal measurement have been used in previous studies, the current study adopted the temporal reproduction task because (1) compared with temporal discrimination, it directly reflects the length of time perception and does not require the use of memory to maintain the temporal anchors; (2) compared with verbal estimation, it does not require semantic processing to translate temporal information into words; and (3) it have been observed to be sensitive to both arousal and attention (Grondin, 2010; Gil and Droit-Volet, 2011; Rattat and Droit-Volet, 2012; Ogden et al., 2014). We only recruited heterosexual participants without social anxiety because sexual preference and social anxiety both can affect time perception (Samson and Janssen, 2014; Jusyte et al., 2015). Moreover, differences in distinctiveness between attractive and unattractive faces were accounted for, as it might influence time perception (Ogden, 2013).

## Experiment 1

### Methods

#### Participants

Twenty men and 20 women were recruited for this study. This sample size is consistent with that of a previous study (Ogden, 2013), and an *a priori* power analysis indicated a sample of 24 would have adequate power (1–β ≥ 0.80) to detect a medium effect, $\eta_{p}^{2}$ = 0.06 (Faul et al., 2007). Participants ranged in age from 18 to 29 (mean ± SD = 21.20 ± 3.21) years. All participants reported themselves to be Chinese, heterosexual, right-handed, and to have normal or corrected-to-normal vision. The Chinese version of the Liebowitz Social Anxiety Scale (He and Zhang, 2004) showed that all participants’ scored below 30, indicating they had no social anxiety (He and Zhang, 2004).

#### Apparatus and Materials

A PC with a 17″ LCD screen (1,024 × 768 pixels, 60 Hz) and a keyboard was used to present stimuli and record data via E-Prime 1.1 (Psychological Software Tools, Pittsburgh, PA, United States).

Given individual differences in esthetic standards, the stimuli for each participant were based on the participant’s selection from an image pool. This image pool, which was specifically made for the current study by the authors, consisted of 24 color images of Chinese faces (12 male and 12 female). All faces had a neutral expression; the head faced the camera directly; the eyes looked directly into the camera; and no ornamentation was present. According to qualitative estimates provided by seven experts with experience in facial attractiveness studies, these images were discernible in gender, heterogeneous in attractiveness, and homogeneous in age. These images were standardized to a size of 320 × 400 pixels with a white background. We did not standardize skin color or skin blemishes because they are known to be components of attractiveness (Fink et al., 2006). Participants were instructed to rate the attractiveness and distinctiveness of each face on a 9-point Likert scale, and to select 4 attractive female faces, 4 attractive male faces, 4 unattractive female faces, and 4 unattractive male faces as their personalized stimuli. Kendall’s coefficient of concordance showed a moderate inconsistency in participants’ selections, *W* = 0.44, *p* < 0.001, suggesting that individual differences in facial esthetic do exist; as such, using personalized stimuli was deemed appropriate. A repeated-measures ANOVA over attractiveness scores, with Attractiveness (attractive vs. unattractive) and Facial gender (male vs. female) as within-subjects factors, revealed a significant main effect of Attractiveness, *F*(1,39) = 2049.33, *p* < 0.001, $\eta_{p}^{2}$ = 0.98. The scores were systematically lower for the unattractive images than for the attractive images, *p* < 0.001. Neither the main effect of Facial gender (*p* = 0.75) nor the interaction between Attractiveness and Facial gender (*p* = 0.83) was significant, suggesting that the manipulation of attractiveness was effective. In addition, another repeated-measures ANOVA using the same design for distinctiveness scores showed that the main effects of Attractiveness (*p* = 0.92) and Facial gender (*p* = 0.53) were not significant, and neither was the interaction between Attractiveness and Facial gender (*p* = 0.87), suggesting that the distinctiveness of faces was matched (Table 1). For the Materials, see Supplementary  Materials and Task (Data Sheet 3).

**TABLE 1:** Mean scores (standard deviation) of attractiveness and distinctiveness for the faces in Experiment 1.

	**Attractiveness**		**Distinctiveness**	
	**Male face**	**Female face**	**Male face**	**Female face**
Attractive face	7.71 (0.57)	7.67 (0.50)	5.18 (0.68)	5.10 (0.80)
Unattractive face	2.55 (0.52)	2.54 (0.68)	5.15 (0.75)	5.11 (0.76)

#### Procedure

The experiment lasted approximately 35 min and consisted of two phases. Phase 1 was dedicated to selection of the participant’s personalized stimuli, and Phase 2 aimed to measure their time perception using a temporal reproduction task. Participants were seated in a quiet and well-lit room at a distance of approximately 60 cm from the screen, which subtended less than 16° of the horizontal and vertical visual angles, for the duration of the experiment. The participants gave their written informed consent prior to the experiment. The local ethics committee of Southwest University approved the experimental protocol.

Phase 1 consisted of three parts. First, the 24 facial images in the image pool were presented to the participants three times in a random order to familiarize the participants with the whole image pool. The participants were then instructed to rate the attractiveness and distinctiveness of each face on a 9-point Likert scale ranging from “not at all” to “extremely.” Finally, the participants were asked to select 4 attractive female faces, 4 attractive male faces, 4 unattractive female faces, and 4 unattractive male faces from among the 24 facial images for use as their personalized stimuli.

**FIGURE 1:**
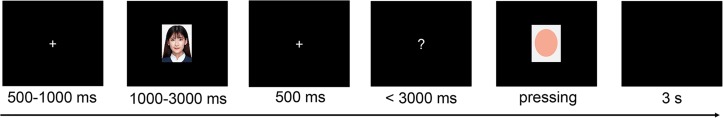
Schematic illustration of the temporal reproduction task.

Phase 2 began with the provision of detailed instructions that informed the participant of the nature of the temporal reproduction task. Following this, each trial began with the presentation of a fixation lasting between 500 and 750 ms. This was immediately followed by a facial image, which was presented for a variable duration of 1,000, 1,500, 2,000, 2,500, or 3,000 ms. Subsequently, a question mark appeared on the computer screen cueing the participant to reproduce the duration of the facial image. This remained on the screen either for 3,000 ms or until the participant responded by pressing the space bar for a duration equivalent to the amount of time the facial image was presented. An image of a pink oval with a white background appeared at the center of the screen at the beginning of the key press and remained on the screen until the participant released the space bar, Figure 1. For the Materials, see Supplementary Materials and Task (Data Sheet 3). To ensure that the procedure was consistent for each participant, all 24 images from the original pool were presented once for each duration, for a total of 120 experimental trials. The unselected images were used as fillers; therefore, the data recorded for them were excluded from further analysis. Five practice trials (in which one additional facial image of each duration was presented) were completed at the beginning of the task to clarify the instructions and to familiarize participants with the task. At the end of Phase 2, the participants were asked to respond to the question “How closely did you follow the instructions given to you regarding reproducing the duration of each facial image?” Responses were given on a 9-point Likert scale ranging from “not at all” to “completely.”

#### Statistics

A procedure based on previous studies was applied to control for outliers (Chang et al., 2011; Rammsayer and Verner, 2014). First, we checked whether the ratings given for self-reported compliance with the duration reproduction task were equal to or lesser than 5. However, all the rating scores ranged from 7 to 9, and the mean score (8.38 ± 0.77) was significantly higher than was the midpoint (i.e., 5), *t*(38) = 27.76, *p* < 0.001; thus, none of the participants were excluded according to the first criterion. Second, all reproduced durations that were more than ±2 SDs from the mean for each condition were considered invalid trials. Of all trials, 0.94% were removed from the data pool based on this criterion. Third, each participant’s remaining reproduced durations were submitted to a one-way ANOVA with Objective duration (1,000, 1,500, 2,000, 2,500, and 3,000 ms) as the within-subjects factor. The lack of a significant main effect as well as any non-significant differences among the five levels would imply an individual’s inability to reproduce the durations. None of the participants were excluded according to this final criterion.

Statistical analysis was performed using SPSS Statistics 20.0 (IBM Corp., Armonk, NY, United States). The significance level was set at 0.05. A four-way repeated-measures ANOVA was performed on the average reproduced durations, with Objective duration (1,000, 1,500, 2,000, 2,500, and 3,000 ms), Attractiveness (attractive and unattractive), and Facial gender (male and female) as within-subjects factors, and Participant gender (male and female) as a between-subjects factor. *Post hoc* testing of the main effects was conducted using the Bonferroni method. Significant interactions were analyzed using simple effects models. Partial η-squared ($\eta_{p}^{2}$) was reported as a measure of effect size.

### Results

**FIGURE 2:**
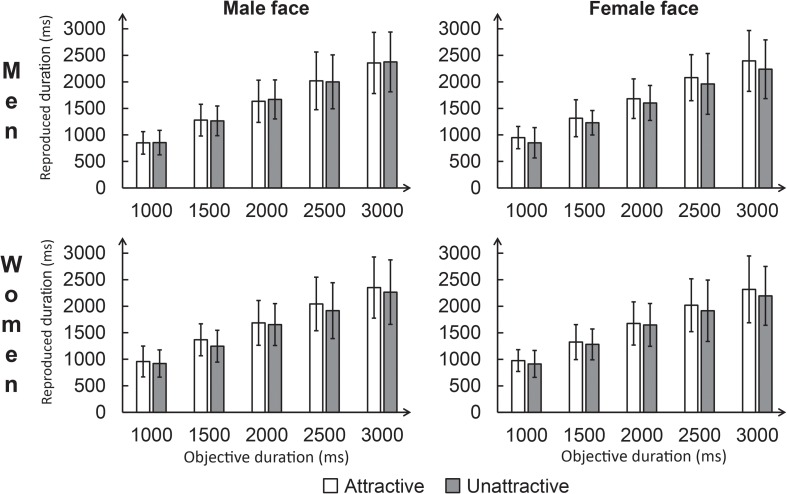
Average reproduced durations for each condition in Experiment 1. The error bar represents the standard deviation.

The four-way ANOVA revealed a significant main effect of Objective duration, *F*(4,152) = 285.89, *p* < 0.001, $\eta_{p}^{2}$ = 0.88, *post hoc* analysis showed that the reproduced durations increased along with increases in objective durations and were significantly different from each other, *p*s < 0.001. The main effect of Attractiveness was also significant, *F*(1,38) = 16.02, *p* < 0.001, $\eta_{p}^{2}$ = 0.30, and was modulated by Facial gender, as revealed by a significant interaction between Attractiveness and Facial gender, *F*(1,38) = 6.22, *p* < 0.05, $\eta_{p}^{2}$ = 0.14. A three-way interaction between Attractiveness, Facial gender, and Participant gender was also significant, *F*(1,38) = 8.33, *p* < 0.01, $\eta_{p}^{2}$ = 0.18. No other significant main effects or interactions were observed, *p*s > 0.20. The average durations reproduced in each condition are presented in Figure 2.

We further analyzed the three-way interaction by dividing it in terms of participant gender. Among men, a repeated-measures ANOVA was conducted for the reproduced durations, with Attractiveness (attractive vs. unattractive) and Facial gender (male vs. female) as within-subjects factors. The main effect of Facial gender was not significant, *p* = 0.98. A significant main effect of Attractiveness was found, *F*(1,19) = 4.47, *p* < 0.05, $\eta_{p}^{2}$ = 0.19, which was modulated by facial gender, as revealed by a significant interaction between these factors, *F*(1,19) = 13.90, *p* < 0.01, $\eta_{p}^{2}$ = 0.42. The simple effects analysis showed that in the attractive face condition, the average reproduced duration was longer for female faces (1683.95 ± 347.65 ms) than for male faces (1628.10 ± 350.43 ms), *p* < 0.01. The average reproduced duration was shorter for female faces (1576.26 ± 350.95 ms) than for male faces (1632.78 ± 347.33 ms) in the unattractive face condition, *p* < 0.01. Additionally, the average reproduced duration was longer for attractive faces than for unattractive faces in the female face condition, *p* < 0.01. In contrast, the average reproduced duration did not significantly differ between attractive faces and unattractive faces in the male face condition, *p* = 0.88.

Referring to women, a repeated-measures ANOVA was conducted over reproduced durations using the same design as male participants. The results showed a significant main effect of Attractiveness, *F*(1,19) = 13.66, *p* < 0.01, $\eta_{p}^{2}$ = 0.42, such that reproduced durations were systematically shorter for the unattractive faces (1595.57 ± 374.49 ms) than for the attractive faces (1672.04 ± 379.73 ms), *p* < 0.01. The main effect of Facial gender (*p* = 0.24) and the interaction between attractiveness and facial gender (*p* = 0.78) failed to reach significance. For the raw data, see Supplementary Data Sheet 1.

In summary, the results of Experiment 1 showed that women’s time perception when viewing attractive faces is longer than that when viewing unattractive faces, regardless of face gender, and that this effect of facial attractiveness was only observed in men when they viewed female faces. In other words, the effect of facial attractiveness on time perception showed a gender difference. However, since Experiment 1 required participants to rate and select the stimuli before they completed the temporal task, participants might have been aware of the experimental purpose. Awareness of time distortions has been found to regulate time perception (Droit-Volet et al., 2015). Moreover, our participants were exposed to the stimuli multiple times before the temporal task, and repeated exposure to a stimulus has been shown to increase its attractiveness (Peskin and Newell, 2004). Consequently, to avoid the potential influence of awareness and exposure, we added another experiment in which the rating and selection of stimuli was performed after the temporal task.

## Experiment 2

### Methods

#### Participants

Another 20 men and 20 women were recruited for this experiment. Participants ranged in age from 18 to 25 (20.85 ± 1.87) years. All participants reported themselves to be Chinese, heterosexual, right-handed, and to have normal or corrected-to-normal vision. Their scores on the Liebowitz Social Anxiety Scale were all below 30, indicating they had no social anxiety (He and Zhang, 2004).

#### Apparatus and Materials

The apparatus and materials were the same as in Experiment 1.

Kendall’s coefficient of concordance calculated from participants’ stimuli selection indicated a moderate inconsistency in selection among participants, *W* = 0.46, *p* < 0.001, suggesting that the use of personalized stimuli was appropriate. The repeated-measures ANOVA examining attractiveness scores, with Attractiveness (attractive vs. unattractive) and Facial gender (male vs. female) as within-subjects factors, revealed a significant main effect of Attractiveness, *F*(1,39) = 1604.86, *p* < 0.001, $\eta_{p}^{2}$ = 0.98. The scores were systematically lower for unattractive images than for attractive images, *p* < 0.001. Neither the main effect of Facial gender (*p* = 0.18) nor the interaction between Attractiveness and Facial gender (*p* = 0.30) was significant, suggesting that the manipulation of attractiveness was effective. Another repeated-measures ANOVA for distinctiveness scores, using the same design, showed that the main effects of Attractiveness (*p* = 0.99) and Facial gender (*p* = 0.39) were not significant, and their interaction was also not significant (*p* = 0.37), suggesting that the distinctiveness of the facial stimuli did not differ (Table 2).

**TABLE 2:** Mean scores (standard deviations) of attractiveness and distinctiveness for the faces in Experiment 2.

	**Attractiveness**		**Distinctiveness**	
	**Male face**	**Female face**	**Male face**	**Female face**
Attractive face	7.45 (0.69)	7.48 (0.59)	4.66 (0.57)	4.77 (0.93)
Unattractive face	2.69 (0.61)	2.91 (0.62)	4.89 (0.98)	4.78 (0.83)

#### Procedure

The procedure was the same as in Experiment 1, except that the selection task (Phase 1) came after the temporal task (Phase 2). The participants gave their written informed consent before the experiment. The local ethics committee of Southwest University approved the experimental protocol.

#### Statistics

The method of controlling outliers was the same as in Experiment 1. None of the participants were excluded after checking their self-reported compliance ratings, as their scores ranged from 7 to 9 and the mean score (8.55 ± 0.50) was significantly higher than the midpoint (i.e., 5), *t*(39) = 44.56, *p* < 0.001. Furthermore, 0.81% of all trials were removed from the data according to the second criterion (i.e., their reproduced durations exceeded ±2 SDs from the mean). None of the participants were excluded based on the final criterion because the results of the one-way ANOVAs indicated that all participants followed the instructions to reproduce the target durations.

The statistical analysis method was the same as that in Experiment 1.

### Results

**FIGURE 3:**
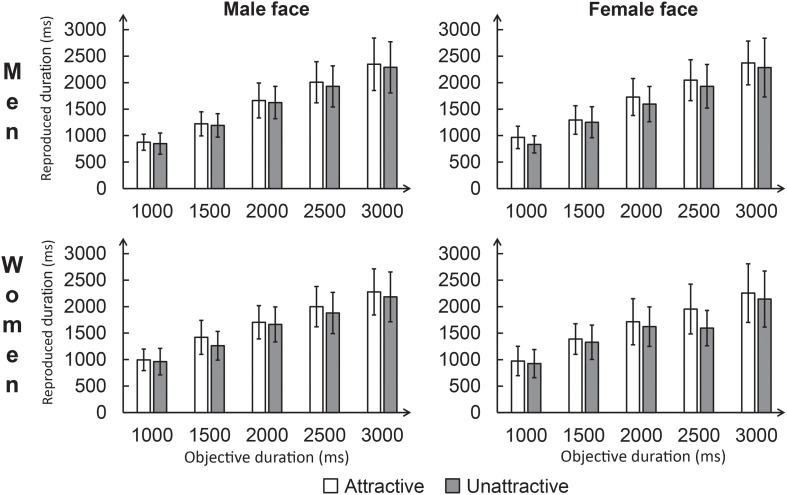
Average reproduced durations for each condition in Experiment 2. The error bar represents the standard deviation.

A repeated-measures ANOVA was conducted for reproduced durations, with Objective duration (1,000, 1,500, 2,000, 2,500, and 3,000 ms), Attractiveness (attractive and unattractive), and Facial gender (male and female) as within-subjects factors, and Participant gender (male and female) as a between-subjects factor. The findings revealed a significant main effect of Objective duration, *F*(4,152) = 325.02, *p* < 0.001, $\eta_{p}^{2}$ = 0.90, and *post hoc* analysis showed that the reproduced durations significantly increased with the objective duration, *p*s < 0.001. The main effect of Attractiveness was also significant, *F*(1,38) = 57.58, *p* < 0.001, $\eta_{p}^{2}$ = 0.60, and was modulated by Facial gender and Participant gender, as revealed by a significant three-way interaction, *F*(1,38) = 5.78, *p* < 0.05, $\eta_{p}^{2}$ = 0.13. No other significant main effects or interactions were observed, *p*s > 0.06. The average durations reproduced in each condition were presented in Figure 3.

To further analyze the significant three-way interaction, we performed separate analyses according to Participant gender. Among men, a repeated-measures ANOVA was conducted for reproduced durations, with Attractiveness (attractive vs. unattractive) and Facial gender (male vs. female) as within-subjects factors. The main effect of Facial gender was not significant, *p* = 0.28. However, a significant main effect of Attractiveness was found, *F*(1,19) = 45.16, *p* < 0.001, $\eta_{p}^{2}$ = 0.70, which was modulated by Facial gender, as revealed by a significant interaction between these factors, *F*(1,19) = 6.57, *p* < 0.05, $\eta_{p}^{2}$ = 0.26. The analysis of the simple effects showed that in both the male and female face conditions, the average reproduced duration was longer for attractive (male: 1623.11 ± 268.81 ms; female: 1681.53 ± 287.67 ms) faces than for unattractive faces (male: 1576.58 ± 278.91 ms; female: 1579.23 ± 306.11 ms), *p*s < 0.01. However, in the attractive face condition, the average reproduced duration was longer for female faces than for male faces, *p* < 0.05. The average reproduced duration for female faces was not significantly different from that for male faces in the unattractive face condition, *p* = 0.93.

Among women, we conducted another repeated-measures ANOVA using the same design as for men. The results showed a significant main effect of Attractiveness, *F*(1,19) = 22.37, *p* < 0.001, $\eta_{p}^{2}$ = 0.54, such that the reproduced durations were systematically shorter for the unattractive faces (1585.26 ± 317.99 ms) than for the attractive faces (1668.12 ± 310.94 ms), *p* < 0.01. Neither the main effect of Facial gender (*p* = 0.47) nor the interaction between attractiveness and facial gender (*p* = 0.51) was significant. For the raw data, see Supplementary Data Sheet 2.

The results of Experiment 2 showed that women’s time perception for both attractive male and female faces was systematically longer than was that for unattractive faces. Among men, however the effect of facial attractiveness on time perception was greater when they viewed female faces than when they viewed male faces. To summarize, Experiment 2 confirmed that the gender factor is able to modulate the effect of facial attractiveness on time perception.

## Discussion

The aim of the current study was to examine whether gender modulates the effect of facial attractiveness on time perception. Using a temporal reproduction task, Experiment 1 measured participants’ time perception of faces of different attractiveness levels and gender after completing a facial attractiveness selection task. To avoid the potential influence of task order, Experiment 2 set the selection task after the temporal task. In line with our hypothesis, both experiments showed that participant gender modulates the effect of facial attractiveness on time perception, such that men and women showed inconsistent performance for time perception when viewing same-sex faces.

In the current study, the manipulation of attractiveness was carried out based on individual participants’ personal esthetic standards. This represents a salient difference from most previous studies. Previous researchers selected stimuli based on average ratings of attractiveness, with the same stimuli being presented to every participant (Arantes et al., 2013; Ogden, 2013; Tomas and Španić, 2016). Instead, we required participant to choose stimuli that they considered to be attractive and unattractive. Although there is a set of some esthetic standards for faces that is common across almost all humans, some aspects of these esthetic standards may differ among people as a result of their biological, psychological, behavioral, and social backgrounds (Cunningham et al., 1995; Kou et al., 2013). Therefore, the current method of manipulating attractiveness might be more effective than previous methods, especially for the current small-sample study.

Furthermore, we excluded or controlled for several salient potential influences on the extent to which facial attractiveness could affect time perception. Ogden (2013) stated that facial distinctiveness is an important factor in how facial attractiveness affects time perception. That is, unattractive faces may cause people to divert their attention away from the passage of time because unattractive faces are more distinctive and atypical than are attractive faces. However, the current study observed that facial attractiveness has influence on time perception in the absence of significant differences in the distinctiveness, suggesting that the effect of facial attractiveness on time perception may be independent of facial distinctiveness. Arantes et al. (2013) presented faces with smiling and neutral expressions as stimuli and observed that facial attractiveness affects time perception, so their results might have arisen from a confound between facial attractiveness and expression, as smiling expressions usually induce the perception of a longer display time compared to neutral expressions (Gil and Droit-Volet, 2012). To avoid such confounds, the stimuli presented in the current study consisted only of faces with neutral expressions. Furthermore, social anxiety has been shown to lead to the perception of longer display times for faces (Jusyte et al., 2015), and sexual preference may modulate arousal and attention to same- and opposite-sex faces (Samson and Janssen, 2014), thereby influencing time perception. We therefore recruited heterosexual participants without social anxiety to avoid the potential influences of these factors and observe the independent effect of facial attractiveness on time perception. Although there are some differences in methods between the current study and previous studies, we have replicated to some extent the previous findings that attractive faces for women induced the perception of a longer display time than did unattractive faces (Arantes et al., 2013; Ogden, 2013; Tomas and Španić, 2016). These findings suggest that the effect of facial attractiveness on women’s time perception is repeatable and stable.

Importantly, our results revealed the important role played by gender in the effect of facial attractiveness on time perception. Specifically, women exhibited a longer time perception for attractive faces than for unattractive faces, regardless of facial gender. For men, on the other hand, time perception for attractive female faces was longer than that for unattractive female faces, while the effect of attractiveness on time perception for male faces was smaller (Experiment 2) or did not even reach significance (Experiment 1).

As mentioned in the introduction, the temporal dilating effect can be explained by the critical role played by arousal in speeding up the internal clock. Previous studies have found that both men and women may be aroused more by attractive than by unattractive opposite-sex faces (Liang et al., 2010; Paulmann et al., 2013). In contrast, a gender difference between men and women to same-sex faces has been found. That is, arousal can be induced in women by both same- and opposite-sex attractive faces (Hazlett and Hoehn-Saric, 2000), but there is little evidence that facial attractiveness affects men’s arousal in response to same-sex faces. According to SET, the increased arousal is associated with an accelerated internal clock, and may result in a longer time perception (Gibbon et al., 1984; Lake et al., 2016). Therefore, it is logical to infer that the arousal mechanism contributes significantly to the gender difference in the temporal dilation effect of attractiveness.

However, the temporal dilating effect can be also explained by an increase in the amount of attentional resources allocated to timing. Previous researchers found that although attractive faces capture attention faster than do unattractive faces, men show a stronger bias toward attractive opposite-sex faces than do women (Sui and Liu, 2009; Valuch et al., 2015). According to SET, capturing attention earlier would result in fixed additive timing. However, the contribution of additive timing to dilating time perception would decrease with the increase in objective duration. Since we failed to observe any interaction between Attractiveness and Objective duration, we think that the additive timing effect of attention contributes little to gender differences in the temporal dilation effect of attractiveness. One explanation may lie in the stimulus presentation time. Previous studies that have observed a significant effect of additive timing mainly used subsecond stimuli (Mattes and Ulrich, 1998; Enns et al., 1999; Tse et al., 2004), whereas the current study used suprasecond stimuli. Thus, the dilating effect of additive timing might have been overwhelmed by the long stimulus presentation time.

Some researchers found that it is easier for opposite-sex attractive faces to hold the attention of both men and women than for opposite-sex unattractive faces as a result of evolutionary instincts (Valuch et al., 2015; Li et al., 2016; Xu et al., 2016). In contrast, when viewing same-sex faces, attractive faces were found to be more effective in capturing women’s attention compared to unattractive faces (Maner et al., 2003; Valuch et al., 2015). A similar effect was not observed in men (Maner et al., 2003). These findings indicate that facial attractiveness affects attention, and may distract the attention allocated to timing. However, we did not observe a temporal dilation effect for relatively unattractive to attractive faces. Thus, attention might play a weak role in gender differences with the temporal dilation effect of attractiveness. It should be noted that as we did not manipulate arousal or attention, so these inferences require further verification. Moreover, these inferences may not be extended to the studies that used subsecond timescales, because the timescales of the current study were suprasecond, and previous studies have shown the subsecond and suprasecond durations are processed using different mechanisms (Pouthas et al., 2005; Lewis and Miall, 2006; Hayashi et al., 2014; Murai and Yotsumoto, 2016).

Several limitations of the present study and directions for future studies should be noted. First, all the male facial images presented in the current study were without facial hair. Facial hair, which is a male secondary sexual characteristic, signals underlying health, age, and social dominance in men, thereby enhancing their attractiveness to women (Dixson et al., 2016). Future researchers could examine whether secondary sexual characteristics, such as facial hair, modulate the effect of facial attractiveness on time perception, which would further advance understanding of these gender differences. Second, we compared only attractive with unattractive faces and did not include a neutral condition. Thus, we do not know whether attractive and unattractive faces were perceived as longer, shorter or the same for neutral faces. Future research should include a neutral condition to improve this limitation. Third, although our results and the results of previous studies demonstrated that facial attractiveness affects the time perception, few studies have explored the mechanism underlying this effect. Although most studies have inferred that arousal and attention are important determinants of time perception, inconsistent inferences have been made about how they operate in this role. Therefore, future research should directly explore these mechanisms. Fourth, the type of temporal task can influence the temporal effect (Gil and Droit-Volet, 2011; Ogden et al., 2014). Discrimination, production, verbal estimation, and reproduction are four main tasks used in temporal research studies (Grondin, 2010; Mioni et al., 2016). The reproduction task adopted in the current study has been found to be more sensitive to attention than is the discrimination task (Baudouin et al., 2006; Rattat and Droit-Volet, 2012), requires more working memory to maintain temporal information than does the production task (Mioni et al., 2016), and does not require additional semantic processing to translate temporal information for verbal estimation (Ogden et al., 2014). Therefore, the repeatability of the current study might depend on task type and it should be verified under different methodological conditions.

## Conclusion

In conclusion, the current study provides evidence that gender modulates the effect of facial attractiveness on time perception. When viewing opposite-sex faces, both men and women perceived time to last longer for attractive faces relative to unattractive faces; however, when viewing same-sex faces, women still perceived the time as longer in the case of attractive faces, while the effect of facial attractiveness on men’s time perception tended to decrease. These findings provide evidence of how gender influences how facial attractiveness affects time perception.

## Author Contributions

YT, HY, and XH designed the experiment. YT and LL acquired and analyzed the data. All authors contributed to the interpretation of the data and approved the final version of the manuscript.

## Conflict of Interest Statement

The authors declare that the research was conducted in the absence of any commercial or financial relationships that could be construed as a potential conflict of interest.
