# Supplementary figures and images for: Gender Differences in the Effect of Facial Attractiveness on Perception of Time
Source: Front Psychol. 2019 Jun 4;10:1292. doi: 10.3389/fpsyg.2019.01292 (PMC6558225; doi:10.3389/fpsyg.2019.01292)

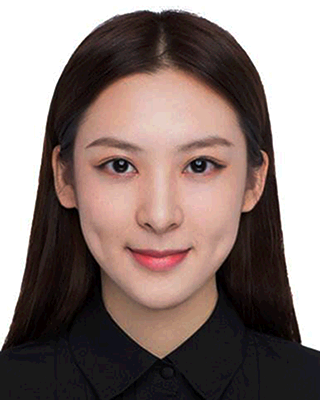

Supplement: Supplementary file 3 [file Data_Sheet_3.zip › duration reproduction task/1.bmp]

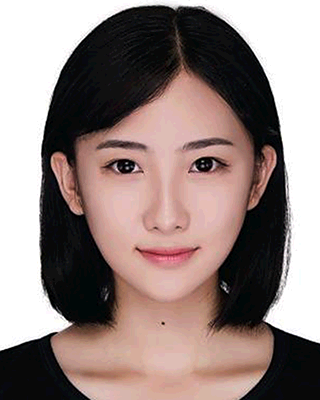

Supplement: Supplementary file 3 [file Data_Sheet_3.zip › duration reproduction task/2.bmp]

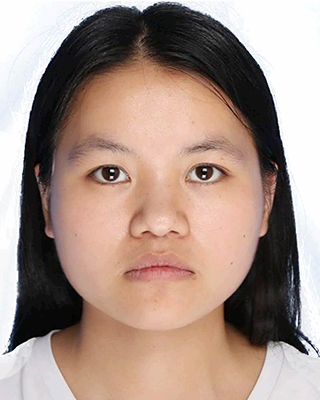

Supplement: Supplementary file 3 [file Data_Sheet_3.zip › duration reproduction task/21.bmp]

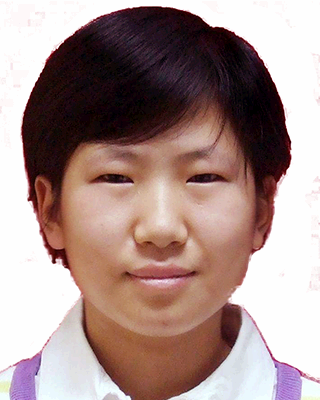

Supplement: Supplementary file 3 [file Data_Sheet_3.zip › duration reproduction task/22.bmp]

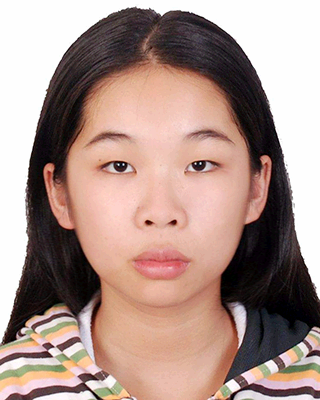

Supplement: Supplementary file 3 [file Data_Sheet_3.zip › duration reproduction task/23.bmp]

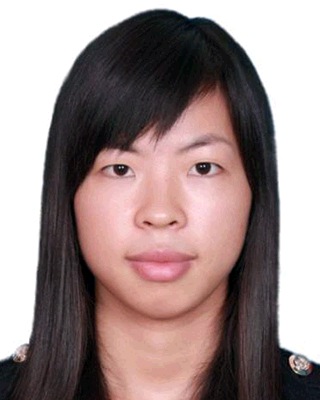

Supplement: Supplementary file 3 [file Data_Sheet_3.zip › duration reproduction task/24.bmp]

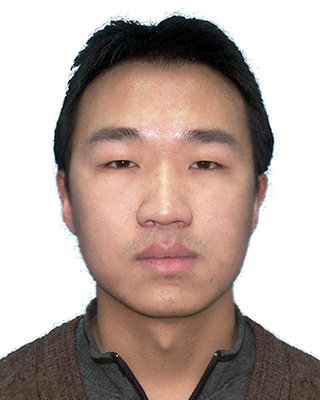

Supplement: Supplementary file 3 [file Data_Sheet_3.zip › duration reproduction task/25.bmp]

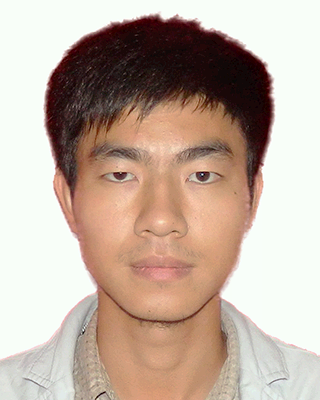

Supplement: Supplementary file 3 [file Data_Sheet_3.zip › duration reproduction task/26.bmp]

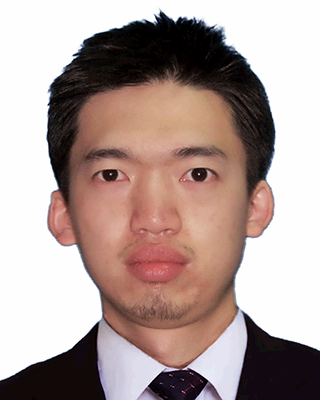

Supplement: Supplementary file 3 [file Data_Sheet_3.zip › duration reproduction task/27.bmp]

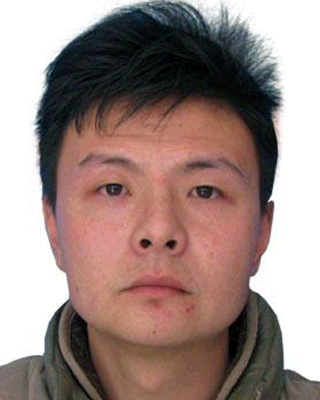

Supplement: Supplementary file 3 [file Data_Sheet_3.zip › duration reproduction task/28.bmp]

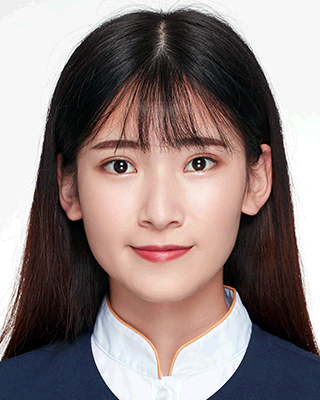

Supplement: Supplementary file 3 [file Data_Sheet_3.zip › duration reproduction task/3.bmp]

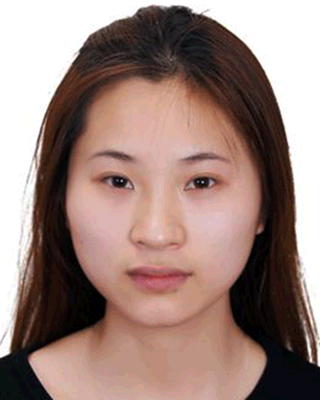

Supplement: Supplementary file 3 [file Data_Sheet_3.zip › duration reproduction task/31.bmp]

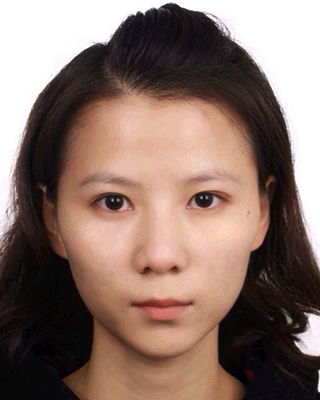

Supplement: Supplementary file 3 [file Data_Sheet_3.zip › duration reproduction task/32.bmp]

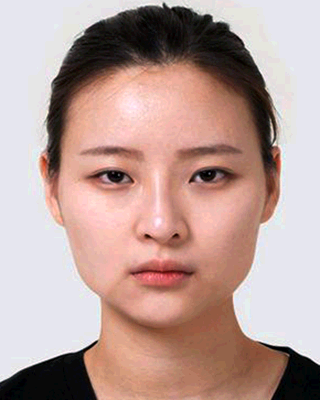

Supplement: Supplementary file 3 [file Data_Sheet_3.zip › duration reproduction task/33.bmp]

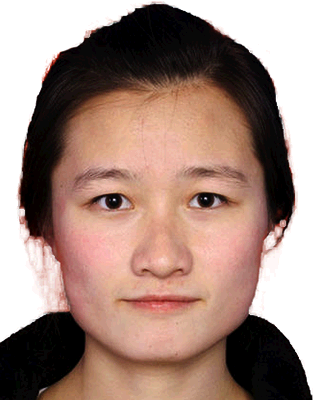

Supplement: Supplementary file 3 [file Data_Sheet_3.zip › duration reproduction task/34.bmp]

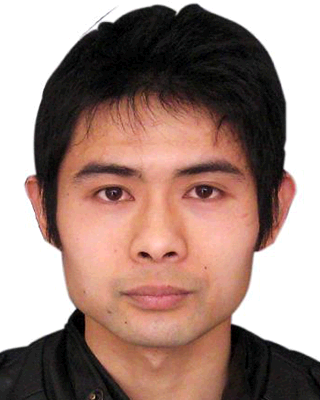

Supplement: Supplementary file 3 [file Data_Sheet_3.zip › duration reproduction task/35.bmp]

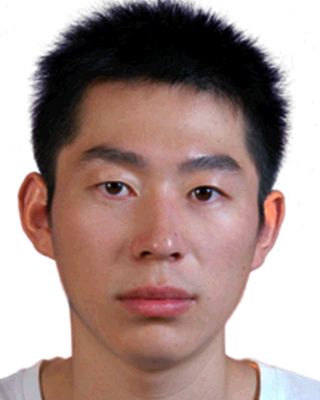

Supplement: Supplementary file 3 [file Data_Sheet_3.zip › duration reproduction task/36.bmp]

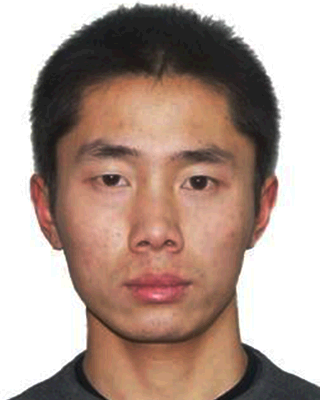

Supplement: Supplementary file 3 [file Data_Sheet_3.zip › duration reproduction task/37.bmp]

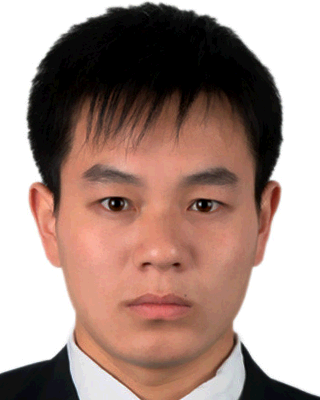

Supplement: Supplementary file 3 [file Data_Sheet_3.zip › duration reproduction task/38.bmp]

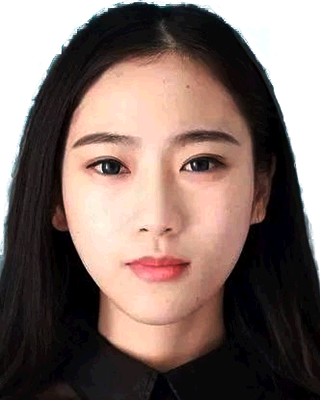

Supplement: Supplementary file 3 [file Data_Sheet_3.zip › duration reproduction task/4.bmp]

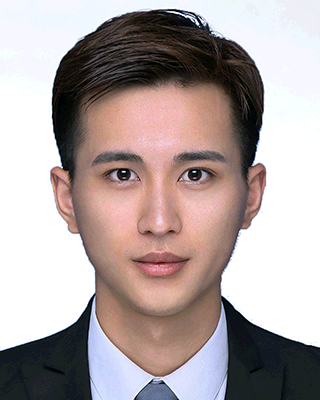

Supplement: Supplementary file 3 [file Data_Sheet_3.zip › duration reproduction task/5.bmp]

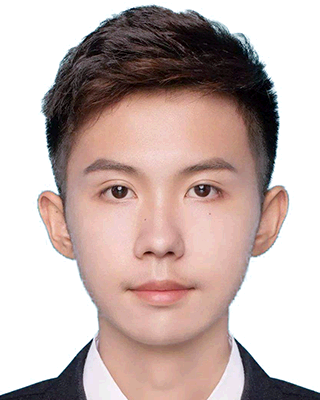

Supplement: Supplementary file 3 [file Data_Sheet_3.zip › duration reproduction task/6.bmp]

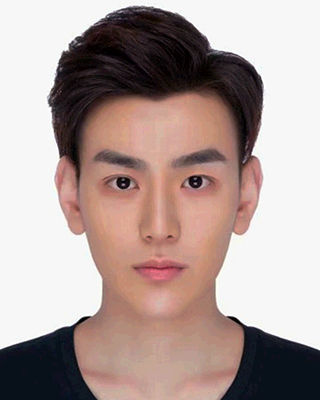

Supplement: Supplementary file 3 [file Data_Sheet_3.zip › duration reproduction task/7.bmp]

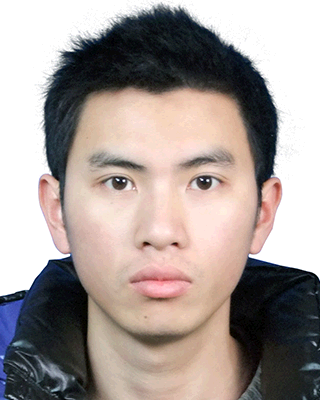

Supplement: Supplementary file 3 [file Data_Sheet_3.zip › duration reproduction task/8.bmp]

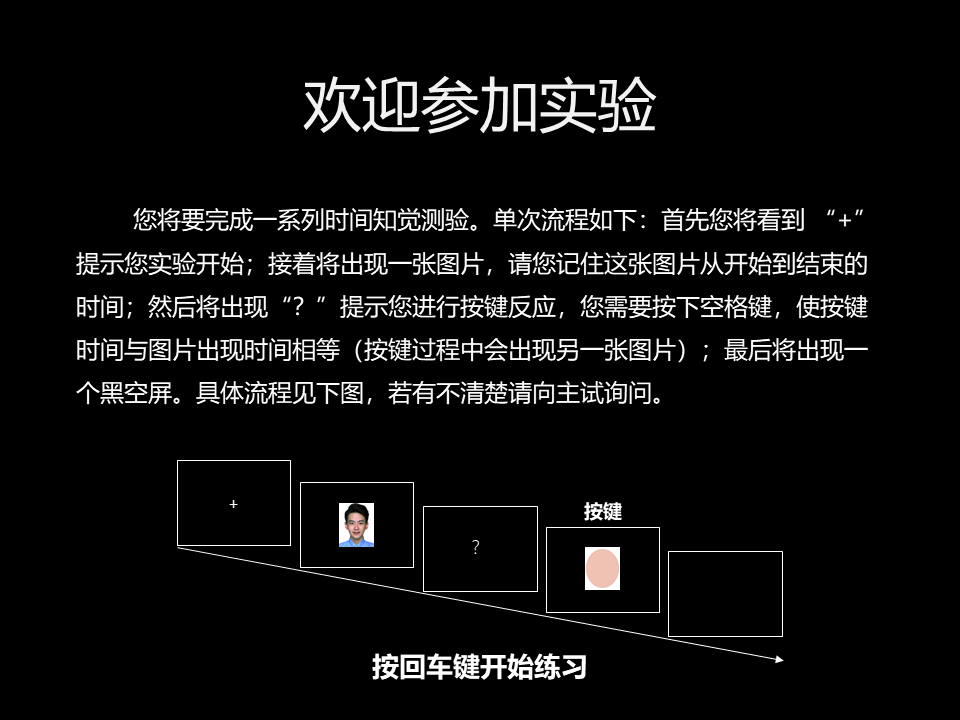

Supplement: Supplementary file 3 [file Data_Sheet_3.zip › duration reproduction task/i1.BMP]

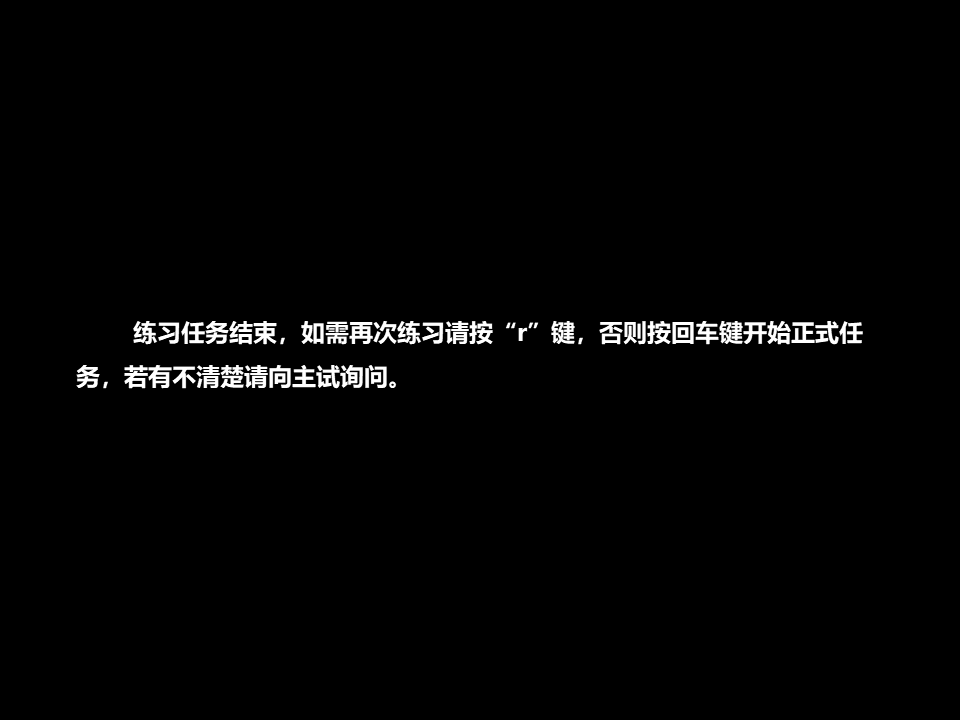

Supplement: Supplementary file 3 [file Data_Sheet_3.zip › duration reproduction task/i2.BMP]

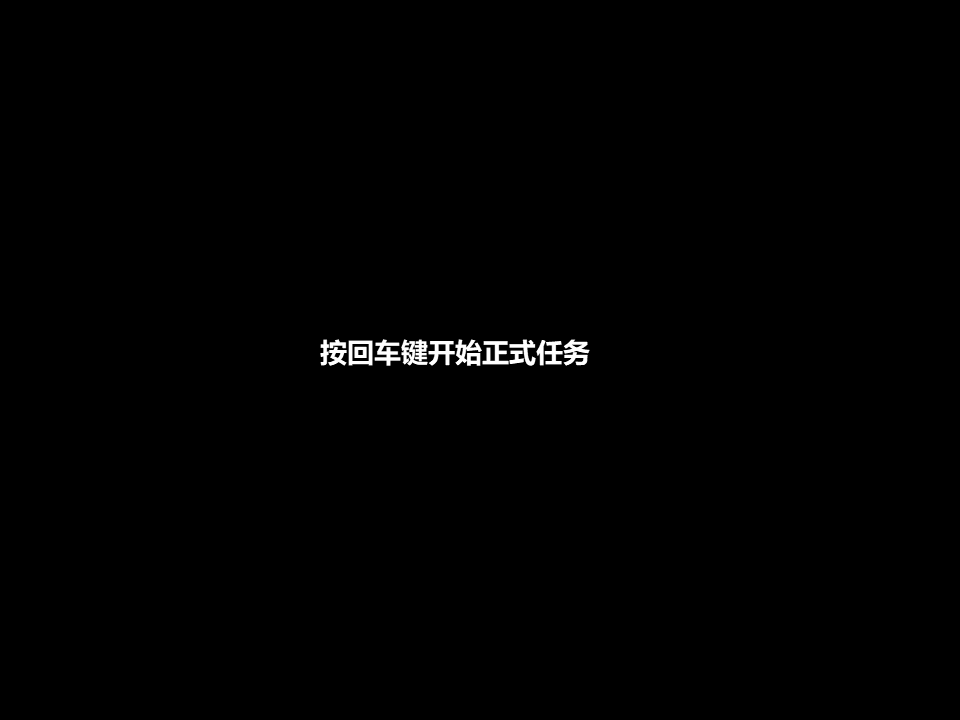

Supplement: Supplementary file 3 [file Data_Sheet_3.zip › duration reproduction task/i3.bmp]

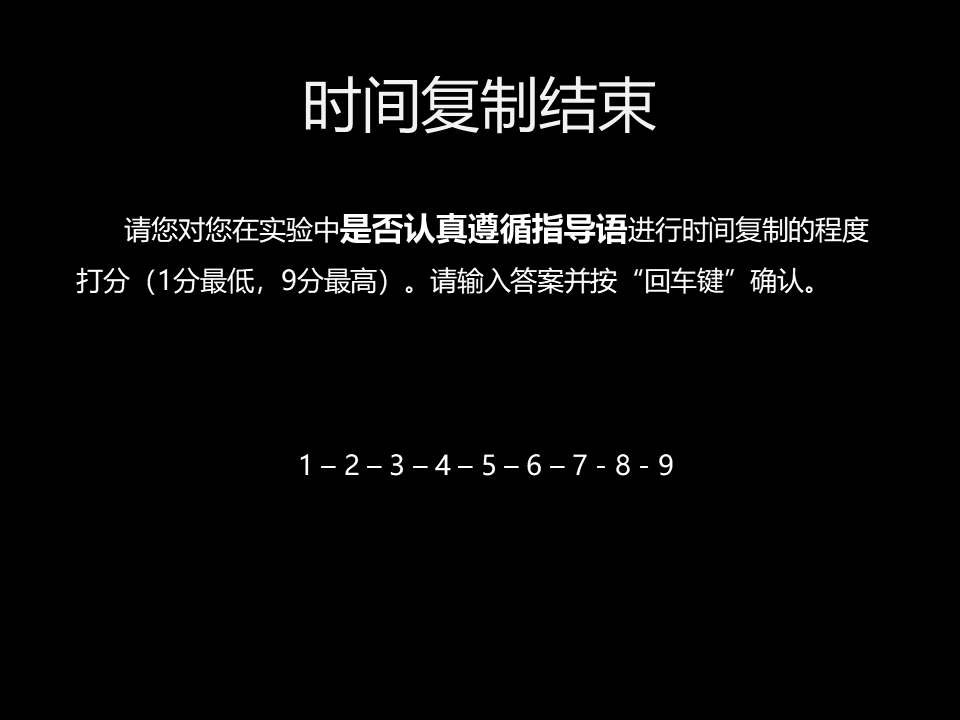

Supplement: Supplementary file 3 [file Data_Sheet_3.zip › duration reproduction task/i6.BMP]

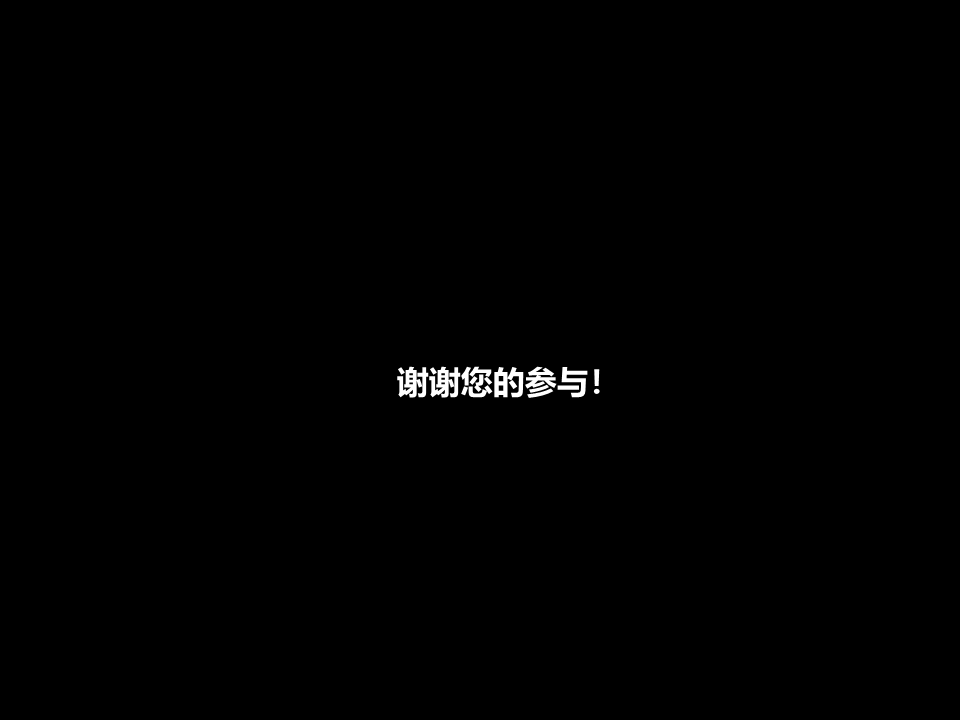

Supplement: Supplementary file 3 [file Data_Sheet_3.zip › duration reproduction task/i7.BMP]

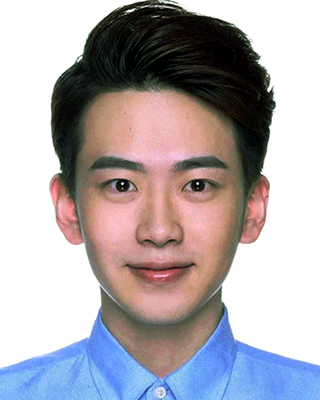

Supplement: Supplementary file 3 [file Data_Sheet_3.zip › duration reproduction task/learnpic.bmp]

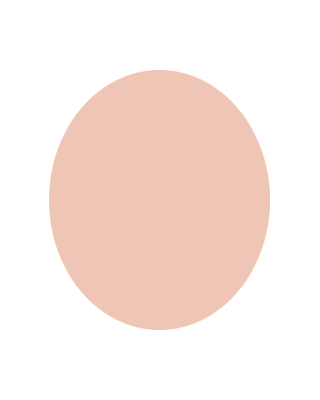

Supplement: Supplementary file 3 [file Data_Sheet_3.zip › duration reproduction task/pinkface.bmp]
